# Impact of near work on perceived stress according to working hours: The Korea National Health and Nutrition Examination Survey VI (2013–2015)

**DOI:** 10.1371/journal.pone.0204360

**Published:** 2018-10-17

**Authors:** Inchul Jeong, Yun-Sik Cho, Kyung-Jong Lee, Jae Bum Park

**Affiliations:** 1 Department of Occupational and Environmental Medicine, Ajou University School of Medicine, Suwon, Korea; 2 Department of Occupational and Environmental Medicine, Ajou University Hospital, Suwon, Korea; Universidad Miguel Hernandez de Elche, SPAIN

## Abstract

The purpose of this study was to investigate the relationships among working hours, near work time, and perceived stress. In total, data of 3,776 workers from the Korea National Health and Nutrition Examination Survey VI were examined. The workers’ working hours per week, daily near work time, and complaints of perceived stress were analyzed in conjunction with other sociodemographic and occupation-related variables. Multivariate logistic analysis found that workers with 3 and ≥4 hours of near work were more likely to report high perceived stress than were the reference group who had <1 hour per day of near work, with odds ratios (ORs) (95% confidence intervals [CIs]) of 1.34 (1.01–1.78) and 1.94 (1.53–2.46), respectively. Additionally, those working 50 and more hours a week were more likely to report high perceived stress with ORs of 1.51 (1.19–1.90) and 1.88 (1.42–2.48), respectively. When stratified by working hours, workers with daily near work time of ≥4 hours were more likely to report high perceived stress with ORs of 2.21 (1.45–3.37), 2.27 (1.30–3.97), and 3.47 (1.80–6.69), among the workers with 40–49, 50–59, and ≥60 weekly working hours, respectively. Workers with greater near work time are at risk for high perceived stress. Moreover, this risk was found to be higher among workers with longer working hours. Therefore, work cycle modification and reductions in near work time are necessary to prevent stress-related health outcomes.

## Introduction

The negative health impacts of long working hours are an important issue and are especially pressing in Korea, since it is one of the countries with the longest working hours.[1] Previous studies have reported an association between long working hours and medical conditions such as coronary heart diseases, sleep problems, depression, and injuries at work.[2–4] Although the mechanism differs by diseases category, it is suggested that psychological stress plays an important role in the development of such diseases. Therefore, reducing workers’ psychological stress levels is important in the prevention of work-related diseases.

Previous research has found a relationship between long working hours and psychological stress. Some studies reported that long working hours are related to the stress response,[5, 6] and another reported a relationship between long working hours and elevated cortisol concentration, which is a response of hypothalamic-pituitary-adrenal axis activation under stress stimulation.[7] Moreover, recent studies have consistently suggested that the relationship between long working hours and several medical conditions or lifestyle behavior, such as atrial fibrillation, type 2 diabetes, coronary heart diseases, stroke, anxiety, depression, and alcohol use, can be partially explained by psychological stress.[8–12] However, it has also been suggested that long working hours *per se* are not as strongly related to psychological stress as are workload and amount of work.[6, 13] Therefore, it is important to investigate which type or condition of work causes greater mental load on workers, since most previous studies have only considered total working hours as a risk factor.

Near work is a type of work with short viewing distance, such as reading or computer work, and it is becoming more common in industrialized countries with the increased use of video display terminals (VDTs), such as computers, tablets, and smartphones, during work. However, near work may lead to adverse health problems such as eye problems, including asthenopia and dry eye, and musculoskeletal problems involving the neck and shoulders.[14–16] In previous studies, reduced eye blink and awkward body posture have been suggested as culprits in the relationship between near work and health problems such as eye problems and musculoskeletal problems, respectively.[15, 17] Additionally, psychological stress is regarded as a pathway in the development of such health problems.[16, 18] However, though some studies have reported a relationship between VDT use during work and mental symptoms,[19, 20] few have investigated the direct relationship between near work and psychological stress and the dose-response effect of near work.

Previously, workers’ income insecurity and employers’ desire to reduce cost were suggested as reasons for long working hours.[4] Further, owing to increasing use of computers and smart devices, near work is practically unavoidable in almost all occupations. Therefore, if near work has a synergistic effect on psychological stress along with long working hours, it should be investigated to prevent adverse health outcomes. Hence, the purpose of this study was to investigate the relationships among working hours, near work time, and perceived stress and their dose–response effects in Korean workers. Furthermore, since the relationship between working hours and psychological stress has already been described in previous studies, this study examined the effects of near work time on perceived stress with the stratification of working hours.

## Materials and methods

### Study participants

This study used data from the 6^th^ Korea National Health and Nutrition Examination Survey (KNHANES), which was conducted from 2013 to 2015 by the Korea Centers for Disease Control and Prevention (KCDC) with participation from 22,948 individuals selected through systematic sampling to represent the Korean population. Among the participants, 3,776 paid workers between the ages of 19 and 49 years were included, since near work time has been investigated only for participants in that age range. The survey protocols for the KNHANES VI were approved by the Institutional Review Board (IRB) of the KCDC (IRB No. 2013-07CON-03-4C, 2013-12EXP-03-5C, and 2015-01-02-6C), and informed consent was obtained from all participants.

### Assessment of near work time, working hours, and perceived stress

In the KNHANES, the participants were asked, “How many hours per day on average did you spend on near work (e.g., computer work or reading) in the past year?” They were given four options for response: (1) less than 1 hour, (2) 1–2 hours, (3) 3 hours, and (4) 4 hours or more. Data on the participants’ average weekly working hours in the past year were collected to classify them into four groups: (1) less than 40 hours, (2) 40–49 hours, (3) 50–59 hours, and (4) 60 hours or more. The participants were asked for their perceived stress levels, and four options were given for response: (1) very much, (2) much, (3) a little, and (4) almost none. They were then divided into two groups; those who gave the first two responses were classified as the high stress group, and those who gave the last two were classified as the low stress group.

### Covariates

Demographic factors such as sex, age, education level, and marital status and work-related factors such as occupation, employment type, and shift work were used as covariates. Age groups were 19–29, 30–39, and 40 years or older. Education level was divided into two groups: those with an education level of high school graduate or less, and those with college or above. Occupation was divided into white collar and blue collar based on the Korean Standard Classification of Occupation. Employment types were classified as regular, temporary, and day laborer, and the last two were merged to form a non-regular workers group. Finally, the workers who reported that their work schedule as fixed day work were classified as day workers, and the others were classified as shift workers.

### Statistical analysis

We conducted χ^2^-tests and logistic regression analyses to compare the characteristics of participants according to the level of perceived stress. Odds ratios (ORs) and 95% confidence intervals (95% CIs) for high perceived levels of stress were estimated using multivariate logistic analyses. Additionally, logistic regression analyses stratified by working hours were conducted to identify any difference in the effect of near work time on perceived stress by working hours. All statistical tests were two-tailed, and *p*-values less than 0.05 were regarded as statistically significant. All statistical analyses were conducted with the SAS software package version 9.4 (SAS Institute, Cary, NC, USA).

## Results

In total, 1,188 (31.5%) out of the total of 3,776 workers reported high perceived stress. In the univariate analyses, distributions of age, marital status, occupation, daily near work time, and weekly working hours showed significant differences between the high and low stress groups. Workers aged 40 years or older were less likely to report high levels of stress than those in other age groups. Additionally, unmarried workers, white collar workers, workers with daily near work times of 4 hours or more, and workers with weekly working hours of 60 hours or more were more likely to report high levels of stress. However, the distributions by sex, education level, employment type, and shift work did not show significant differences between the two groups (Table 1). A detailed analysis revealed that the workers aged 40 years or older were less engaged in near work with 25.7% working less than an hour and 42.9% working more than 4 hours; while 7.0% of workers aged 19–29 worked less than an hour, and 64.2% worked more than 4 hours; and 12.8% of workers aged 30–39 worked less than an hour, and 57.5% worked more than 4 hours.

**Table 1:** General characteristics of study participants.

	Perceived stress		*p*-value
	High	Low	
	(*n* = 1,188)	(*n* = 2,588)	
	*N* (%)	*N* (%)	
Sex			
Male	581 (30.7)	1,311 (69.3)	0.335
Female	607 (32.2)	1,277 (67.8)	
Age			
19–29	324 (33.1)	654 (66.9)	<0.001
30–39	469 (34.6)	885 (65.4)	
≥40	395 (27.4)	1,049 (72.7)	
Education level			
High school or less	344 (29.5)	822 (70.5)	0.090
College or above	844 (32.3)	1,766 (67.7)	
Marital status			
Yes	761 (30.3)	1,753 (69.7)	0.029
No	428 (33.9)	835 (66.1)	
Occupation			
White collar	944 (32.8)	1,934 (67.2)	0.002
Blue collar	244 (27.2)	654 (72.8)	
Employment type			
Regular	872 (31.4)	1,908 (68.6)	0.865
Non-regular	316 (31.7)	680 (68.3)	
Shift work			
Yes	190 (28.4)	479 (71.6)	0.067
No	998 (32.1)	2,109 (67.9)	
Near work time/day			
<1 hour	141 (23.0)	471 (77.0)	<0.001
1–2 hours	165 (26.2)	464 (73.8)	
3 hours	142 (27.9)	367 (72.1)	
≥4 hours	740 (36.5)	1,286 (63.5)	
Working hours/week			
<40	306 (28.6)	765 (71.4)	<0.001
40–49	507 (29.9)	1,187 (70.1)	
50–59	238 (36.2)	419 (63.8)	
≥60	137 (38.7)	217 (61.3)	

In the logistic analyses, workers with daily near work times of 3 hours showed a significantly higher OR for reporting high levels of stress after adjustment (OR, 95% CI: 1.34, 1.01–1.78) than did those with daily near work times of less than an hour, and workers with daily near work times of 4 hours or more showed significant higher ORs for reporting high levels of stress in both crude (1.92, 1.56–2.37) and adjusted (1.94, 1.53–2.46) models. Additionally, workers working 50–59 (1.42, 1.15–1.75 and 1.51, 1.19–1.90 in the crude and adjusted models, respectively) and 60 hours or more a week (1.58, 1.23–2.03 and 1.88, 1.42–2.48 in the crude and adjusted models, respectively) showed significantly higher ORs for reporting high levels of stress than did those working less than 40 hours a week (Table 2).

**Table 2:** Odds ratios for high perceived stress by near work time and working hours.

	Crude	Adjusted*
	*OR* (95% CI)	*OR* (95% CI)
Near work time/day		
<1 hour	1.00 (Reference)	1.00 (Reference)
1–2 hours	1.19 (0.92–1.54)	1.22 (0.93–1.59)
3 hours	1.29 (0.99–1.69)	1.34 (1.01–1.78)
≥4 hours	1.92 (1.56–2.37)	1.94 (1.53–2.46)
Working hours/week		
<40	1.00 (Reference)	1.00 (Reference)
40–49	1.07 (0.90–1.26)	1.06 (0.87–1.28)
50–59	1.42 (1.15–1.75)	1.51 (1.19–1.90)
≥60	1.58 (1.23–2.03)	1.88 (1.42–2.48)

* Adjusted for sex, age, education level, marital status, occupation, employment type, and shift work

To compare the effects of near work time on perceived stress by working hours, multivariate logistic analyses stratified by working hours were conducted (Table 3). In workers with less than 40 hours of work a week, daily near work time showed no significant effect on perceived stress. However, there were significant effects of near work time on perceived stress in workers with weekly working hours of 40–49, 50–59, and 60 hours or more. In workers with 40–49 hours of work a week, those with daily near work times of 3 hours and 4 hours or more showed significantly higher ORs for reporting high levels of stress (1.81, 1.11–2.95 and 2.21, 1.45–3.37, respectively) than did the reference group. In the workers with 50–59 hours of work a week, those with daily near work times of 4 hours or more showed a significantly higher OR for reporting high levels of stress (2.27, 1.30–3.97). Additionally, in the workers with weekly 60 hours or more of work a week, those with daily near work times of 3 hours and 4 hours or more showed significantly higher ORs for reporting high levels of stress (2.66, 1.23–5.77 and 3.47, 1.80–6.69, respectively). Furthermore, the results for the three working hour groups showed significant relationships in the linear trend tests (*p* <0.001 for the 40–49-hour group, *p* = 0.003 for the 50–59-hour group, and *p* <0.001 for the group working 60 hours or more).

**Table 3:** Adjusted odds ratios for high perceived stress by amount of near work time stratified by working hours.

	Working hours/week			
	<40	40–49	50–59	≥60
	*OR* (95% CI)	*OR* (95% CI)	*OR* (95% CI)	*OR* (95% CI)
Near work time/day				
<1 hour	1.00 (Reference)	1.00 (Reference)	1.00 (Reference)	1.00 (Reference)
1–2 hours	0.88 (0.56–1.38)	1.46 (0.92–2.33)	1.53 (0.81–2.88)	1.46 (0.69–3.06)
3 hours	0.89 (0.55–1.44)	1.81 (1.11–2.95)	0.84 (0.39–1.83)	2.66 (1.23–5.77)
≥4 hours	1.26 (0.84–1.90)	2.21 (1.45–3.37)	2.27 (1.30–3.97)	3.47 (1.80–6.69)

Adjusted for sex, age, education level, marital status, occupation, employment type, and shift work

## Discussion

This study examined the relationship between near work time, working hours, and perceived stress. The results indicate that the workers who were engaged in 3 or more hours of daily near work or in 50 hours or more work a week were more likely to report high levels of stress. Moreover, the relationship between near work time and perceived stress strengthened as the number of working hours increased. To the best of the knowledge of the authors, this is the first study exploring the direct relationship between near work time and psychological stress using large-scale epidemiological data.

Several previous studies have reported a relationship between long working hours and stress,[5, 21, 22] and the results of this study are consistent with this study. Individuals working 50–59 or 60 or more hours per week were more likely to report high levels of stress than those working less than 40 working hours per week. A previous report suggested the mechanism by which long working hours affect work-related stress; long working hours reduce time for sleep, recovery from work, and recreational activities. In addition, they increase job demands and time for exposure to workplace hazards.[4] Therefore, workers with long working hours are more likely to report work-related stress.

On the other hand, few studies have reported a relationship between near work and stress. A study of the relationship between VDT work and visual fatigue using structural equation modeling reported that job stress is partially responsible for visual fatigue in VDT workers. The study reported that job stress among VDT workers is affected by the physical features of VDT and environmental conditions.[16] Other studies reported that increased job demands on VDT workers are related to high levels of perceived stress.[18, 23] However, hours of near work have not been considered a risk factor for psychological stress in such studies. In the present study, having 3 or more hours of near work per day was shown to be related to stress. Karasek’s job demand–control model may be a plausible explanation for the association. According to this model, workers with high job demands and low control are defined as the high strain group, which is more likely to report high levels of job stress.[24] Since some characteristics of near work (e.g., awkward body posture and mental concentration[17, 25]) are regarded as job demand factors in the demand–control model, it is likely that near work might entail increased job demands according to the number of hours worked.

However, there was no significant impact of near work time on perceived stress in workers with short working hours (<40 hours/week). A possible explanation for this is the characteristics of regular workers in this group. In Korea, regular workers with short working hours are more likely to have work autonomy in the workplace; therefore, the relationship between near work time and perceived stress was not significant with ORs (CIs) of 0.65 (0.33–1.30), 0.56 (0.26–1.22), and 0.82 (0.45–1.51) for near work time of 1–2, 3, and 4 hours, respectively. In contrast, the ORs (CIs) were higher in other regular workers (1.42 [0.97–2.09], 1.43 [0.95–2.15], and 2.36 [1.69–3.30] for near work time of 1–2 hours, 3 hours, and 4 hours, respectively). Therefore, although the proportion of regular workers was lower in the workers with short working hours (making up 45.4% of the short working hour group versus a mean of 84.8% for the other groups), the characteristics of regular work may have impacted our results. Additionally, the impact of near work time was significant only for those with 3 or more hours a day. A previous study suggested that there is a threshold effect in the relationship between VDT work and mental symptoms, with no significant effects below 5 hours of daily VDT work.[19] Likewise, this study may also show a threshold of 3 hours of near work a day. Finally, the relationship was stronger among the workers with very long working hours (≥60 hours/week). Previously, it has been suggested that chronic stress and burnout are reciprocally related.[26] Since long working hours are reported to be related to burnout,[27] it is possible that burnout among workers with very long working hours makes them more vulnerable to stress. Hence, the impact of near work time increased with working hours. However, since burnout, which has been suggested as a pathway, was not measured in this study, further investigation to clarify this relationship is needed in future studies.

There are certain strengths in this study. First, it was a large-scale epidemiological study including 3,776 workers, more than 80% of whom are exposed to daily near work time of 1 hour or more. Moreover, the workers were systematically sampled from the Korean population, which allows us to regard the results as representative of the Korean population. Additionally, analyses stratified by working hours were conducted in this study, allowing us to isolate the effect of near work time on perceived stress from that of working hours. However, there are also limitations when interpreting the results of this study. First, owing to the cross-sectional design, we cannot establish whether the association is causal. Additionally, the measurement of the study outcome (perceived stress) was based on a single subjective question. Therefore, future studies with a prospective design and objective outcome measurement will be helpful in determining the causal relationship.

To prevent adverse health problems from near work, work schedule modification is necessary. Previous studies examined and suggested suitable work cycles for VDT work. One study recommended a work/rest cycle of 50/7.5 minutes before noon and 100/15 minutes in the afternoon.[28] Another recommended 15 minutes/micro break to prevent musculoskeletal problems and 30/5 minutes to prevent eye problems.[29] Since such health problems are also affected by psychological stress, following these work cycles would be helpful to reduce stress in workers engaged in near work.

In conclusion, near work time is independently related to perceived stress as well as working hours in Korean workers. The strength of the relationship was greater among workers with longer working hours. Therefore, policies restricting near work time and encouraging appropriate breaks, along with controls on working hours, are needed to manage workers’ work-related stress, preferentially for those with longer working hours, to prevent adverse health outcomes that can result from work-related stress.
